# Hemodynamic and Thrombotic Vulnerability in Pulmonary Arterial Hypertension at High Altitude: Multivariable Predictors of Mortality

**DOI:** 10.3390/medicina62050996

**Published:** 2026-05-20

**Authors:** Rafael Conde-Camacho, Eduardo Tuta-Quintero, Angelica Mora-Barrero, Alirio Bastidas-Goyes, Luis F. Giraldo-Cadavid

**Affiliations:** 1 Department of Pulmonary Hypertension, Fundación Neumológica Colombiana, Bogotá 111321, Colombia; 2 Department of Biosciences, School of Engineering, Universidad de La Sabana, Chía 250001, Colombia; 3 Department of Internal Medicine and Epidemiology, School of Medicine, Universidad de La Sabana, Chía 250001, Colombia; eduardotuqu@unisabana.edu.co (E.T.-Q.); angelicamoba@unisabana.edu.co (A.M.-B.); alirio.bastidas@unisabana.edu.co (A.B.-G.); luisf.giraldo@unisabana.edu.co (L.F.G.-C.); 4 Department of Interventional Pulmonology, Fundación Neumológica Colombiana, Bogotá 111321, Colombia

**Keywords:** pulmonary hypertension, observational study, survival, high altitude

## Abstract

*Background and Objectives*: Pulmonary hypertension (PH) remains associated with substantial mortality despite advances in treatment. Although prognostic factors have been widely described at sea level, their behavior in populations living at high altitude remains insufficiently characterized. This study aimed to identify factors associated with mortality during follow-up in patients with Group 1 PH residing at high altitude. *Materials and Methods*: A retrospective cohort study was conducted including patients with confirmed Group I PH diagnosed by right heart catheterization and treated between 2017 and 2022. Clinical, functional, and hemodynamic variables were analyzed. A penalized logistic regression model using Elastic Net methodology was applied to identify variables associated with five-year mortality. *Results*: A total of 165 patients were included, with a mean age of 41 years (SD 13.93), and 84.2% were women. Among PH etiologies, congenital heart disease was the most frequent cause (50.3%), followed by idiopathic PH (33.3%) and connective tissue disease-associated PH (12.7%). Five-year mortality was 13.3% (22/165). Idiopathic pulmonary hypertension was significantly more frequent among deceased patients compared to survivors (13/22 [59.1%] vs. 42/143 [29.4%], *p* = 0.025). Mortality was associated with acute pulmonary embolism, greater smoking burden, worse functional class, and adverse hemodynamic parameters. In multivariable analysis, acute pulmonary embolism (coefficient 0.196; OR 1.216; 95% CI 1.16–1.27; *p* < 0.001), ESC/ERS risk stratification (coefficient 0.158; OR 1.171; 95% CI 1.08–1.26; *p* < 0.001), pulmonary vascular resistance > 25 wood units (coefficient 0.180; OR 1.198; 95% CI 1.13–1.26; *p* < 0.001), and age ≥ 65 years (coefficient 0.171; OR 1.187; 95% CI 1.10–1.27; *p* < 0.001) were identified as risk factors, while female sex showed a protective effect (coefficient −1.041; OR 0.353; 95% CI 0.33–0.37; *p* < 0.001). *Conclusions*: In patients with Group 1 PH living at high altitude, several clinical, functional, and hemodynamic variables were associated with increased mortality, including acute pulmonary embolism, elevated pulmonary vascular resistance, advanced age, and intermediate-high risk stratification. Female sex was associated with lower mortality.

## 1. Introduction

Pulmonary Hypertension (PH) is a disease that affects pulmonary blood circulation and causes progressive deterioration of the pulmonary vasculature, generally because of different cardiovascular and respiratory disorders [1]. According to the classification established at the Seventh World Symposium on PH, this condition is organized into five groups; among them, Group 1 has the poorest prognosis because of its rapid progression and high mortality when not diagnosed and treated promptly [2]. Its course is characterized by a progressive increase in pulmonary vascular resistance (PVR), leading to right ventricular overload, right heart failure, and a substantial increase in mortality risk [3]. Although its incidence is low, estimated at 2 to 7 cases per million adults per year, it represents a highly relevant clinical problem because of its severity, occurring more frequently in women, although this sex difference tends to diminish with age [2].

Recognition of risk factors has improved initial risk stratification, with particular emphasis on reduced distance covered during the Six-Minute Walk Test (6MWT), increased right atrial pressure, and reduced cardiac index, which are frequently assessed by Echocardiography [4]. The presence of comorbidities such as renal dysfunction, Coronary Artery Disease, Type 2 Diabetes, and Chronic Obstructive Pulmonary Disease is also associated with unfavorable clinical outcomes [3]. In addition, biomarkers such as N-terminal pro–B-type natriuretic peptide levels have demonstrated prognostic utility [5].

These variables used as prognostic factors may behave differently depending on the characteristics of each population, particularly in individuals living at high altitude [6,7,8]. Prolonged exposure to hypoxic conditions typical of these regions may promote faster disease progression, intensify pulmonary vascular dysfunction, and worsen clinical outcomes [9,10,11,12,13]. Because altitude exerts a physiological effect on pulmonary hemodynamics, understanding which variables influence survival in these patients is especially important. Therefore, the present study aims to identify risk factors associated with five-year mortality in patients with Group 1 PH treated at a high-complexity center located at high altitude.

## 2. Materials and Methods

A retrospective observational cohort study was conducted in patients treated at the Fundación Neumológica Colombiana between 2017 and 2022, with confirmed diagnosis of PH Group 1. The reporting of this study conforms to the Strengthening the Reporting of Observational Studies in Epidemiology (STROBE) guidelines [14].

### 2.1. Participants

Patients older than 18 years with confirmed PH diagnosed by right heart catheterization were included, provided they attended the specialized program and permanently resided at altitudes above 2500 m above sea level. Patients with incomplete clinical, functional, or hemodynamic records, as well as those with mixed-etiology PH, were excluded.

### 2.2. Variables

The primary outcome variable was five-year mortality, defined based on vital status recorded in institutional databases. Mortality status was obtained from clinical records and administrative registries. Follow-up time was calculated from the date of diagnosis (or program entry) to death or last available contact. As follow-up duration was not uniform across all patients, the outcome was analyzed as a binary variable based on mortality status within the available follow-up period, which may not fully capture five-year outcomes in all cases. Sociodemographic and identification variables analyzed included age and sex, together with physical examination parameters (anthropometric measurements) and relevant clinical history, including Obstructive Sleep Apnea, Diabetes Mellitus, Hypertension, cardiovascular disease, chronic kidney disease, and liver disease. Clinical variables included dyspnea. Functional variables comprised distance walked and oxygen saturation levels during the 6MWT, pulmonary function testing, and diffusing capacity for carbon monoxide. Biochemical markers included N-terminal pro–B-type natriuretic peptide, while echocardiographic variables included systolic pulmonary artery pressure estimated by transthoracic echocardiography. Hemodynamic variables obtained through right heart catheterization included right atrial pressure, PVR, mixed venous oxygen saturation, and cardiac index.

### 2.3. Data Collection and Processing

Information was obtained from the institutional PH database of the Fundación Neumológica Colombiana program, *NeumoVasc Rexpira*, and complemented through systematic review of medical records and administrative registries from the Colombian national health resources administrator. Follow-up information, including vital status, was updated through institutional databases and national administrative health records.

### 2.4. Bias Control

Considering the retrospective design and the potential risk of selection and information bias, mitigation strategies were implemented. These included standardized training of personnel responsible for data collection and an independent double-verification system performed by different members of the research team to minimize errors in clinical data recording and interpretation.

### 2.5. Statistical Analysis

Categorical variables were summarized using absolute frequencies and percentages, whereas continuous variables were described using mean and standard deviation when normally distributed or median and interquartile range when normality assumptions were not met [15]. Categorical variables were compared using the chi-square test or Fisher’s exact test, as appropriate based on expected cell counts. Continuous variables were analyzed using Student’s *t* test or the Mann–Whitney U test, as appropriate [15]. The relationship between risk factors and mortality was initially assessed through bivariate analysis.

Given the low number of mortality events, which limited the applicability of traditional multivariable models, a penalized logistic regression approach using Elastic Net was applied, combining Ridge and Least Absolute Shrinkage and Selection Operator techniques [15]. This method allowed management of multicollinearity and optimization of variable selection in a setting with multiple candidate predictors and limited outcomes. All candidate predictors considered clinically relevant or significant in bivariate analysis were included in the model. Continuous variables were standardized (mean = 0, standard deviation = 1) prior to model fitting. The optimal values of the tuning parameters alpha and lambda were selected using 10-fold cross-validation, minimizing the mean cross-validated error. Missing data were handled using complete-case analysis, as the proportion of missingness was low and assumed to be random. No formal internal validation beyond cross-validation for parameter tuning was performed. Due to the penalized nature of the Elastic Net model, standard inferential statistics are not directly applicable; therefore, reported odds ratios, confidence intervals, and *p* values should be interpreted as approximate measures of association rather than as conventional maximum likelihood–based inference [15].

All patients meeting inclusion criteria within the PH program of the high-complexity center were included. Given the variability in follow-up time and the limited number of events, the outcome was analyzed as a binary variable, and a time-to-event approach was not implemented. No a priori sample size calculation was performed because the study used a census-based cohort design. Data were organized using Data were organized using Research Electronic Data Capture (REDCap, version 16.1.14; accessed on 18 January 2026) and analyzed with RStudio (version 2026.04.0+526; accessed on 18 January 2026). A *p* value < 0.05 was considered statistically significant.

## 3. Results

### 3.1. Baseline Patient Characteristics

**Figure 1:**
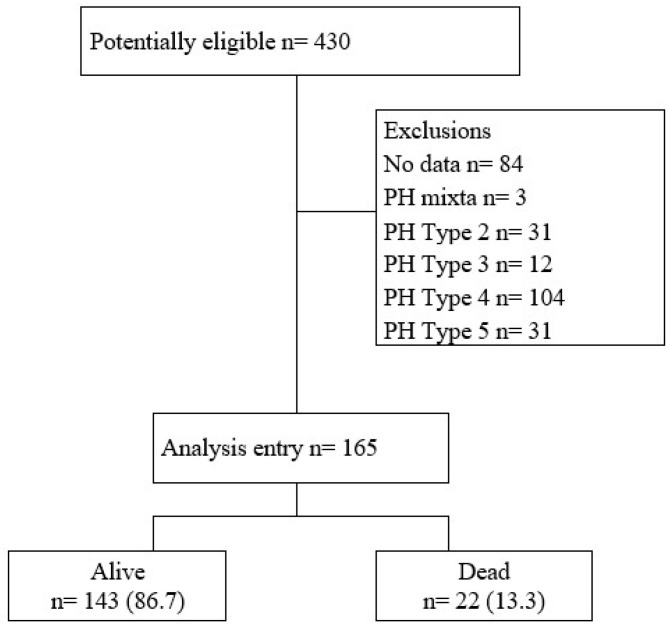
Flowchart of admission to the study. Notes: PH: Pulmonary Hypertension.

Of a total of 430 evaluated patients, 165 were included in the final analysis (Figure 1). Five-year mortality was 13.3% (22/165). The mean age of the cohort was 41 years (SD: 13.93), with female predominance in 84.2% (139/165) of patients. Within the Group 1 PH subclassification, 50.3% (83/165) corresponded to congenital heart disease, 33.3% (55/165) to idiopathic disease, and 12.7% (21/165) to connective tissue disease. Idiopathic PH was significantly more frequent among deceased patients compared to survivors (13/22 [59.1%] vs. 42/143 [29.4%], *p* = 0.025), whereas congenital heart disease was more prevalent among survivors (78/143 [54.5%] vs. 5/22 [22.7%], *p* = 0.050) (Table 1).

Smoking exposure measured as pack-years was significantly higher in deceased patients (12; SD: 11.31) than in survivors (4.3; SD: 4.03) (*p* = 0.002). In addition, acute pulmonary embolism was more frequent among deceased patients (18.2%; 4/22) compared to survivors (4.9%; 7/143) (*p* = 0.020) (Table 2).

When comparing survivors and non-survivors, bosentan use was significantly more frequent among deceased patients (45.5%; 10/22) than among survivors (28.0%; 40/143) (*p* = 0.026). Although not statistically significant, ambrisentan also showed a higher proportion in deceased patients (31.8% vs. 14.7%; *p* = 0.166) (Table 3). The use of sildenafil, macitentan, and other therapies did not differ significantly between groups. Regarding treatment strategies, combination therapy with endothelin receptor antagonist + phosphodiesterase-5 inhibitor was more common among deceased patients (45.5% vs. 33.6%; *p* = 0.276), although this difference did not reach statistical significance.

### 3.2. Functional Status and Hemodynamic Variables

Deceased patients showed worse functional status, reflected by higher St. George’s Respiratory Questionnaire scores compared with survivors (57.1; SD: 21.73 vs. 39.8; SD: 18.22; *p* < 0.001) (Table 4). In the 6MWT, they covered a shorter distance (443.7; SD: 122.7 m vs. 496.3; SD: 102.7 m; *p* = 0.031). Echocardiographic parameters also differed, with lower left ventricular ejection fraction (55.4% vs. 59.3%; *p* = 0.022) and higher pulmonary artery systolic pressure (91.4 mmHg vs. 74.6 mmHg; *p* = 0.014) among deceased patients. Hemodynamically, deceased patients had higher right atrial pressure (14.8 mmHg vs. 10.8 mmHg; *p* = 0.001) and numerically higher mean pulmonary arterial pressure (61.7 vs. 57.8 mmHg; *p* = 0.462) and PVR (16.3 vs. 11.7 WU; *p* = 0.076), although these differences were not statistically significant. Mixed venous oxygen saturation was reduced (61% vs. 67.1%; *p* = 0.005).

### 3.3. Elastic Net Regression Model

Penalized regression analysis identified acute pulmonary embolism (coefficient 0.196; odds ratio [OR] 1.216; 95% CI 1.16–1.27; *p* < 0.001), intermediate-high risk stratification according to European Society of Cardiology/European Respiratory Society guidelines (coefficient 0.158; OR 1.171; 95% CI 1.08–1.26; *p* < 0.001), PVR >2 5 Wood units (coefficient 0.180; OR 1.198; 95% CI 1.13–1.26; *p* < 0.001), and age > 65 years (coefficient 0.171; OR 1.187; 95% CI 1.10–1.27; *p* < 0.001) as the main mortality-associated risk factors. By contrast, female sex was associated with lower mortality risk (coefficient −1.041; OR 0.353; 95% CI 0.33–0.37; *p* < 0.001), suggesting a protective effect (Table 5).

## 4. Discussion

In this study, the main risk factors associated with long-term mortality were identified in patients with Group 1 PH living at high altitude. Predictors of poorer outcome included acute Pulmonary Embolism, intermediate-high risk stratification according to the European Society of Cardiology/European Respiratory Society classification, pulmonary vascular resistance greater than 25 Wood units, higher REVEAL risk score, shorter distance covered during the Six-Minute Walk Test, age above 65 years, and advanced baseline risk classification. In contrast, female sex and higher diastolic blood pressure values were associated with lower mortality. In our cohort, female sex and PH associated with congenital heart disease predominated, whereas the idiopathic form was more frequent among deceased patients. No significant differences in risk factors were identified across the different Group 1 subtypes, reflecting the clinical complexity of this disease and highlighting the importance of integrating clinical, functional, and hemodynamic variables into prognostic assessment.

Identification of prognostic predictors in PH is essential to optimize clinical management and anticipate disease progression. Consistent with previous studies, acute pulmonary embolism in our cohort was associated with a substantial increase in mortality risk [7,8,9,10,16]. Chang et al. reported a significant increase in mortality risk among PH patients who developed pulmonary thromboembolism (hazard ratio: 4.64; 95% confidence interval: 2.74–7.87; *p* < 0.05) [17], which is consistent with our findings. It has been described that patients with pre-existing PH who develop acute pulmonary embolism experience higher mortality than those without PH [7,8], largely because of the higher frequency of right ventricular dysfunction [7,8,9]. From a pathophysiological perspective, pulmonary embolism increases pulmonary vascular resistance through mechanical obstruction of the pulmonary vascular bed and aggravates hypoxemia, triggering hypoxic pulmonary vasoconstriction and release of vasoconstrictive mediators such as serotonin, thromboxane, and histamine, thereby increasing right ventricular afterload [7,8,9,10,11,12]. This acute overload promotes right ventricular dilation, increased wall stress, reduced right coronary perfusion, and, particularly in the presence of systemic hypotension, myocardial ischemia and progressive deterioration of contractility [8,9].

Bogotá, located at 2640 m above sea level, represents a unique pathophysiological environment for patients with Group 1 PH. Chronic exposure to high-altitude hypoxia induces sustained alveolar hypoxemia, which promotes hypoxic pulmonary vasoconstriction, a key mechanism that increases pulmonary arterial pressure and contributes to progressive pulmonary vascular remodeling [18,19]. This process involves endothelial dysfunction, reduced nitric oxide bioavailability, increased endothelin-1 expression, and smooth muscle cell proliferation, all of which are central features in the pathobiology of pulmonary arterial hypertension. In patients with pre-existing Group 1 PH, these hypoxia-driven mechanisms may act synergistically with the underlying disease, accelerating increases in pulmonary vascular resistance and worsening right ventricular afterload [20,21].

In parallel, high-altitude exposure creates conditions favoring a prothrombotic state, including hemoconcentration, increased fibrinogen, platelet activation, elevated factor VIII, dehydration, and reduced mobility [18,19,20,21,22,23]. Chronic hypoxia may also impair right ventricular adaptation by increasing myocardial oxygen demand while simultaneously limiting oxygen delivery, thereby predisposing patients to earlier right ventricular–pulmonary artery uncoupling [18,19,20,24]. Additionally, hypoxia-induced erythrocytosis and increased blood viscosity further contribute to elevated pulmonary vascular resistance and thrombogenicity. In this context, patients with PH may have an increased risk of venous thromboembolism, and when pulmonary embolism occurs, the hemodynamic impact may be particularly severe due to limited right ventricular functional reserve [19,20,21,25].

Chronic hypobaric hypoxia can influence the adaptive capacity of the right ventricle [26,27]. Prolonged exposure to reduced oxygen tension increases myocardial oxygen demand while limiting its supply, which can affect mitochondrial function, myocardial energy, and coronary perfusion [27,28]. In Group 1 PH patients with chronically elevated afterload, these changes can promote earlier uncoupling between the right ventricle and the pulmonary artery, reduced contractile reserve, and maladaptive remodeling [29]. Ultimately, these patients may experience more rapid clinical deterioration and reduced tolerance to pulmonary embolism [26,27,28,29].

From a prognostic perspective, chronic high-altitude exposure may therefore shift the natural history of Group 1 PH toward a more aggressive phenotype, characterized by earlier hemodynamic deterioration and reduced functional reserve [20,21,22,23,24]. This integrated pathophysiological framework may explain the strong association observed in our cohort between mortality and variables reflecting both hemodynamic burden and functional capacity. However, most available evidence derives from healthy populations or individuals without pulmonary arterial hypertension exposed to high altitude; therefore, robust studies specifically addressing Group 1 PH patients living at altitude remain scarce, and age- and altitude-adjusted estimates are still limited [18,19,20,21,22,23,24].

In our cohort, 50% of patients had congenital heart disease, a proportion higher than that reported in industrialized countries and comparable to Latin American registries [18,19,20,21,22]. Some studies conducted in adult congenital heart disease referral centers have reported pulmonary embolism prevalence between 17% and 21% in patients with PH associated with this etiology [18,19]. However, the small sample sizes and methodological heterogeneity limit direct comparison of these findings [18,19,20,21].

Our findings also confirm the prognostic value of stratification tools such as REVEAL and ESC/ERS. Both advanced functional class and shorter distance covered during the six-minute walk test were associated with poorer prognosis, consistent with studies validating REVEAL Lite 2 as a useful tool for clinical assessment [30,31,32,33,34]. Although some reports suggest greater discriminative capacity of REVEAL 2.0 compared with ESC/ERS, both systems remain fundamental for clinical follow-up and therapeutic decision-making [35,36,37]. Taken together, these results support a combined risk stratification strategy.

Pulmonary vascular resistance greater than 25 Wood units was also associated with poorer outcomes. This finding is consistent with reports showing increased mortality with values ≥ 4 Wood units and up to a threefold higher risk when exceeding 7 Wood units in PH secondary to lung disease [38,39,40,41]. In Group 1 PH associated with congenital heart disease, the right ventricle is exposed from early life to sustained pressure and volume overload, favoring progressive adaptation parallel to pulmonary vascular remodeling [37,38,39,40]. This prolonged exposure promotes an adapted right ventricular phenotype characterized by concentric hypertrophy, preserved contractility, and maintenance of ventriculo-arterial coupling, allowing tolerance of very high pulmonary vascular resistance levels over prolonged periods [42,43]. Therefore, these patients may require higher hemodynamic thresholds before right ventricle–pulmonary artery uncoupling and clinical deterioration become evident [44,45]. This pathophysiological context likely explains why PVR greater than 25 Wood units emerged as a mortality predictor in our cohort, whereas substantially lower thresholds have been associated with adverse outcomes in other non-congenital Group 1 PH populations.

From a demographic perspective, mean age was 41 years, with clear female predominance at a 5:1 ratio, consistent with previous studies documenting higher PH prevalence in young women [37,38,39,40]. This difference tends to decrease with age, suggesting a possible influence of hormonal and genetic factors in disease pathophysiology [46,47,48,49]. Manes et al. reported 20-year survival of 87% in patients with Eisenmenger syndrome, higher than that observed in other subgroups, such as corrected defects (87% vs. 36%) [48], which may explain the better survival observed in our cohort among patients with congenital heart disease.

Finally, functional performance also showed important prognostic value. Batal et al. reported significantly shorter six-minute walk distance in patients surviving less than two years compared with those surviving more than five years (244 m vs. 346 m; *p* < 0.001) [50]. Similarly, in our study deceased patients walked shorter distances than survivors (443.7 m vs. 496.3 m; *p* = 0.031), confirming the prognostic value of this test. Unlike the findings of Batal et al., where cardiac index showed no significant association with mortality (*p* = 0.6), our cohort did demonstrate this relationship, which may reflect a more pronounced impact of hemodynamic deterioration in a population chronically exposed to high altitude and potentially greater vulnerability of right ventricular function [50,51].

### Strengths and Limitations

We conducted a retrospective observational study, which introduces the possibility of information bias and misclassification, despite standardized data collection and independent verification of records. The single-center nature of the study, conducted at a high-complexity tertiary referral center, may have introduced referral and selection bias, with a likely overrepresentation of patients with advanced or high-risk disease phenotypes. This could have contributed to the higher observed mortality rates and stronger associations between prognostic variables and outcomes. The identified predictors may primarily reflect a subgroup of patients with more severe Group 1 PH, limiting the generalizability of the findings to broader pulmonary hypertension populations, particularly those treated in non-specialized settings or at lower altitudes.

The duration of follow-up was also heterogeneous among patients, which could have affected the mortality estimate and limits the interpretation of the outcome as a single-point-time measurement. Furthermore, the absence of a comparison cohort residing at low altitude prevents a direct assessment of the independent contribution of chronic high-altitude exposure to disease progression and prognosis. The relatively small sample size and limited number of mortality events may have reduced statistical power, increased the risk of type II error, and contributed to unstable estimates. Although penalized regression methods (Elastic Net) were used to reduce overfitting, some clinically relevant predictors may have been missed [15]. No formal internal or external validation of the predictive model was performed.

Future prospective multicenter studies including patients from different altitudes, particularly low-altitude comparator populations, are needed to validate these findings and better define the specific contribution of chronic hypobaric hypoxia to pulmonary vascular remodeling, right ventricular adaptation, and long-term outcomes in Group 1 PH. In addition, future studies could help determine whether exposure to high altitudes identifies a distinct clinical phenotype that requires risk stratification and altitude-specific management strategies.

## 5. Conclusions

In this cohort of patients with Group 1 PH living at high altitude, several clinical, functional, and hemodynamic variables were associated with increased mortality, including acute pulmonary embolism, elevated PVR, advanced age, and intermediate-high risk stratification. Female sex was associated with lower mortality. These findings underscore the importance of comprehensive assessment and risk stratification in this population; however, given the observational design and absence of a comparison group at lower altitude, no altitude-specific prognostic effect can be established, and the results should be interpreted as associations rather than causal relationships.

## Figures and Tables

**Table 1:** Baseline Characteristics of the Patients.

	Total Population n = 165	Alive n = 143	Dead n = 22	*p* Value
Age years, m (SD)	41.3 (13.93)	41.07 (13.7)	43 (15.64)	0.547
Female, n (%)	139 (84.2)	125 (87.4)	14 (63.6)	0.004
BMI kg/m^2^, m (SD)	25.13 (5.42)	25.19 (5.55)	24.77 (4.59)	0.734
Systolic blood pressure mmHg, m (SD)	113.3 (10.8)	113.6 (11.1)	111.8 (8.71)	0.465
Diastolic blood pressure mmHg, m (SD)	71.7 (10.73)	72.3 (8.95)	68.2 (18.49)	0.314
Group 1 Pulmonary Hypertension Subtypes, n (%)				
Idiopathic	55 (33.3)	42 (29.4)	13 (59.1)	0.025
Hereditary	2 (1.2)	2 (1.4)	(0)	0.839
Connective Tissue	21 (12.7)	17 (11.9)	4 (18.2)	0.441
HIV Infection	1 (0.6)	1 (0.7)	(0)	0.886
Portal Hypertension	1 (0.6)	1 (0.7)	(0)	0.886
Congenital Heart Disease	83 (50.3)	78 (54.5)	5 (22.7)	0.050
Calcium Channel Responders	2 (1.2)	2 (1.4)	(0)	0.839
REVEAL risk stratification, n (%)				
Low risk	99 (60)	94 (65.7)	5 (22.7)	0.0153
Intermediate risk	39 (23.6)	31 (21.7)	8 (36.4)	0.187
High risk	27 (16.4)	18 (12.6)	9 (40.9)	0.002
ESC/ERS risk stratification, n (%)				
Low risk	126 (76.4)	115 (80.4)	11 (50)	0.017
Intermediate risk	38 (23)	28 (19.6)	10 (45.5)	0.128
High risk	1 (0.6)	(0)	1 (4.5)	0.018

Notes: Continuous variables are presented as mean (standard deviation) and categorical variables as number (percentage). BMI = body mass index; HIV = human immunodeficiency virus; ESC/ERS: European Society of Cardiology and European Respiratory Society; REVEAL: Registry to Evaluate Early and Long-Term Management of Pulmonary Arterial Hypertension.

**Table 2:** Comorbidities.

	Total Population n = 165	Alive n = 143	Dead n = 22	*p* Value
Smoking exposure (pack-years), mean (SD)	6.8 (7.17)	4.3 (4.03)	12 (11.31)	0.002
Sleep apnea–hypopnea syndrome, n (%)	12 (7.3)	9 (6.3)	3 (13.6)	0.217
Cancer, n (%)	1 (0.6)	1 (0.7)	(0)	0.884
Diabetes mellitus, n (%)	8 (4.8)	8 (5.6)	(0)	0.634
Systemic hypertension, n (%)	22 (13.3)	17 (11.9)	5 (22.7)	0.164
Chronic kidney disease, n (%)	2 (1.2)	2 (1.4)	(0)	0.832
COPD, n (%)	2 (1.2)	1 (0.7)	1 (4.5)	0.125
Autoimmune disease, n (%)	25 (15.2)	22 (15.4)	3 (13.6)	0.831
Pulmonary embolism, n (%)	2 (1.2)	2 (1.4)	(0)	0.832
Acute pulmonary embolism, n (%)	11 (6.7)	7 (4.9)	4 (18.2)	0.020
Thrombophilia, n (%)	2 (1.2)	1 (0.7)	1 (4.5)	0.125
Congenital Heart Disease Classification, n (%)				
Eisenmenger syndrome	24 (28.2)	21 (26.6)	3 (50)	0.298
Persistent systemic-to-pulmonary shunt	20 (23.5)	19 (24.1)	1 (16.7)	0.719
Small defect	18 (21.2)	17 (21.5)	1 (16.7)	0.803
Post-repair congenital heart disease	23 (27.1)	22 (27.8)	1 (16.7)	0.612

Notes: Continuous variables are presented as mean (standard deviation) and categorical variables as number (percentage). Smoking exposure is expressed in pack-years. COPD = chronic obstructive pulmonary disease.

**Table 3:** Pulmonary arterial hypertension therapies by survival status.

	Total Population n = 165	Alive n = 143	Dead n = 22	*p* Value
Bosentan, n (%)	50 (30.3)	40 (28)	10 (45.5)	0.026
Ambrisentan, n (%)	28 (17)	21 (14.7)	7 (31.8)	0.166
Macitentan, n (%)	17 (10.3)	15 (10.5)	2 (9.1)	0.069
Sildenafil, n (%)	86 (52.1)	73 (51)	13 (59.1)	0.627
Tadalafil, n (%)	3 (1.8)	3 (2.1)	(0)	0.804
Treprostinil, n (%)	6 (3.6)	5 (3.5)	1 (4.5)	0.810
Epoprostenol, n (%)	2 (1.2)	2 (1.4)	(0)	0.839
Iloprost, n (%)	8 (4.8)	6 (4.1)	2 (9)	0.535
Monotherapy, n (%)	28 (17)	23 (16.1)	5 (22.7)	0.440
ERA + PDE5i, n (%)	58 (35.2)	48 (33.6)	10 (45.5)	0.276
ERA + PDE5i + iPROS, n (%)	8 (4.8)	6 (3.4)	2 (9)	0.319
ERA + PDE5i + pPROS, n (%)	8 (4.8)	8 (5.5)	(0)	0.661

Notes: ERA = Endothelin Receptor Antagonist; PDE5i = Phosphodiesterase-5 inhibitor; iPROS/pPROS = inhaled/parenteral prostanoids.

**Table 4:** Baseline functional variables.

	Total Population n = 165	Alive n = 143	Dead n = 22	*p* Value
NYHA, m (DS)	2.3 (0.8)	2.2 (0.79)	2.8 (0.73)	0.600
St. George Score total score, m (SD)	42.1 (19.58)	39.8 (18.22)	57.1 (21.73)	<0.001
6MWT meters, m (SD)	489.3 (106.67)	496.3 (102.68)	443.7 (122.7)	0.031
FVC % predicted, m (SD)	3.124 (0.77)	3.074 (0.67)	3.416 (1.2)	0.194
FEV_1_ % predicted, m (SD)	2.5 (0.66)	2.5 (0.6)	2.6 (0.93)	0.655
DLCO % predicted, m (SD)	24.7 (7.11)	24.8 (6.97)	23.3 (8.19)	0.339
VA % predicted, m (SD)	4.4 (0.9)	4.4 (0.87)	4.7 (1.09)	0.127
Echocardiogram				
Left ventricular ejection fraction %, m (SD)	58.8 (7.45)	59.3 (7.03)	55.4 (9.29)	0.022
Pulmonary artery systolic pressure mmHg, m (SD)	77.1 (30.04)	74.6 (29.77)	91.4 (28.31)	0.014
Right atrial volume mL, m (SD)	38 (25.41)	38.5 (26.09)	31.7 (15.31)	0.084
TAPSE mm, m (SD)	14.7 (10.27)	14.9 (9.79)	13.2 (13.17)	0.452
Right Heart Catheterization				
Mean pulmonary arterial pressure mmHg, m (SD)	58.3 (23.44)	57.8 (23.59)	61.7 (22.64)	0.462
Pulmonary vascular resistance WU, m (SD)	12.3 (8.98)	11.7 (8.42)	16.3 (11.49)	0.076
Pulmonary capillary wedge pressure mmHg, m (SD)	12.2 (4.28)	12.2 (4.35)	12.4 (3.85)	0.886
Right atrial pressure mmHg, m (SD)	11.2 (5.46)	10.8 (5.28)	14.8 (5.59)	0.001
Cardiac output (thermodilution) L/min, m (SD)	2.7 (1.02)	2.8 (1.04)	2.1 (0.52)	<0.001
Cardiac output (Fick method) L/min, m (SD)	2.8 (0.98)	3 (1.02)	2.3 (0.58)	<0.001
Cardiac index L/min/m^2^, m (SD)	4.5 (1.58)	4.6 (1.62)	3.7 (1.01)	0.001
Mixed venous oxygen saturation %, m (SD)	66.5 (9.61)	67.1 (9.51)	61 (9.12)	0.005
NT-proBNP pg/mL, m (SD)	1007 (1053.86)	1037.5 (1101.86)	702 (224.86)	0.001

Notes: Continuous variables are presented as mean (standard deviation). NYHA = New York Heart Association functional class; 6MWT = six-minute walk test; FVC = forced vital capacity; FEV_1_ = forced expiratory volume in 1 s; DLCO = diffusing capacity of the lung for carbon monoxide; VA = alveolar volume TAPSE = tricuspid annular plane systolic excursion; NT-proBNP = N-terminal pro–B-type natriuretic peptide.

**Table 5:** Factors Associated with Mortality.

	Coefficients	Odds Ratio	95% CI	*p* Value
Acute pulmonary embolism	0.196	1.216	1.16–1.27	<0.001
Pulmonary vascular resistance > 25 UW	0.180	1.198	1.13–1.26	<0.001
Age > 65 years	0.171	1.187	1.10–1.27	<0.001
ESC/ERS risk stratification	0.158	1.171	1.08–1.26	<0.001
REVEAL 2.0 risk stratification	0.086	1.090	0.71–1.65	0.686
Baseline St. George’s score	0.021	1.021	0.03–28	0.990
Female sex	−1.041	0.353	0.33–0.37	<0.001

Notes: ESC/ERS: European Society of Cardiology and European Respiratory Society; REVEAL: Registry to Evaluate Early and Long-Term Management of Pulmonary Arterial Hypertension.

## Data Availability

The database used in this study can be found in the hospital informatics system and archives and is not available for public access.
